# Disparate access to nutritional food; place, race and equity in the United States

**DOI:** 10.1186/s40795-021-00434-2

**Published:** 2021-06-29

**Authors:** Garett Sansom, Bryce Hannibal

**Affiliations:** 1 Department of Environmental and Occupational Health, Texas A&M School of Public Health, 1266 TAMU, College Station, TX 77843 USA; 2 Bush School of Government and Public Service, Texas A&M University, 4220 TAMU, College Station, TX 77843 USA

**Keywords:** Food desert, Nutrition, Minority, Obesity, Equity

## Abstract

**Background:**

Prior research has demonstrated minority communities have fewer options to access healthy foods when compared to their majority counterparts. While much focus has been placed upon community-level resources, little research has been placed on the efforts that minority groups need to undergo to reach well-stocked stores to purchase healthy food options.

**Methods:**

As part of the Water, Energy, Food Nexus Research Group at Texas A&M University, a nationally representative survey (*n* = 1612) was conducted to acquire self-reported distance, time, and motives that certain populations must travel to purchase food for themselves and their families.

**Results:**

Findings suggest that minority populations consider saving money, driving less, having a better selection of foods, and have the ability to buy organic foods as an important factor when choosing where to buy foods. Further, minority populations across the nation need to drive a significantly greater (*p* < 0.05) amount of time to reach their destinations than white populations.

**Conclusion:**

This underscores the importance, and scope of the issues, of promoting and implementing more equitably distributed opportunities to purchase healthy food options throughout the United States.

**Supplementary Information:**

The online version contains supplementary material available at 10.1186/s40795-021-00434-2.

## Background

The unequal access to affordable high-quality food within the United States has been highlighted in recent years. In 2013, there were an estimated 17.5 million households that were food insecure with a disproportionate burden placed upon African American and Hispanic homes [1]. Further, disparate rates of obesity and obesity-related illness among minority majority communities, in comparison to majority neighborhoods, have been conclusively shown [2, 3]. The primary explanation for these differences is the existence of “food deserts” within marginalized communities throughout the United States [4]. Food deserts, to be understood as a geographic region in which it is difficult to purchase affordable high-quality food, has been empirically shown to exist primarily in minority neighborhoods [5, 6]. While much research on access to supermarkets have involved utilizing directories and census data [7, 8], food store assessments [7, 9], and Geographic Information Systems (GIS) [10, 11], little work has been conducted using a nationally representative questionnaire. The importance of creating an environment that is conducive to living a healthy lifestyle cannot be overstated and access to quality food is a vital facet in that calculation.

The World Health Organization (WHO) has declared that obesity is one of the most serious public health issues in the twenty-first century. While access to high-quality food is scarce in African American communities, calorie dense fast-food restaurants are in abundance. A food density study showed that neighborhoods with an 80% or greater proportion of African American residents had an average of 2.4 restaurants/mile^2^ compared to 1.5 restaurants/mile^2^ in neighborhoods with only 20% African American residents [12]. This reality has led to Hispanic and Non-Hispanic Black communities shouldering the highest levels of obesity in the United States [13].

Another barrier to equitable food access is costs associated with purchasing groceries. A Seattle-based obesity study [14] conducted a survey (*n* = 1682) on self-reported obesity rates and socioeconomic status variables as well as stratified supermarkets by price of goods and distance to participants. It was discovered that an inverse relationship existed between price and obesity levels, indicating that access to affordable nutritional foods is difficult for many individuals.

Rural communities have also been shown to provide fewer locations to access healthy foods for local residents, however, rural residents that are higher income and predominantly white often have the means to travel farther distances to access more nutritional options [2]. Little research has addressed, at a national level, (1) the time spent traveling to supermarkets and local grocery stores by race (2) the reasoning for traveling to specific locations, be it financial or quality of product, and (3) if rural minority populations in rural locations shoulder an unfair burden in accessing nutritional food compared to their rural white counterparts. This research seeks to close the gap on these research questions.

## Methods

### Survey

As part of the Water, Energy, Food (WEF) Nexus Research Group at Texas A&M University, a pan-university multi-year research endeavor, a nationally representative survey was administered in August 2015. The survey aimed to determine a variety of environmental and WEF issues including access to supermarkets and grocery stores, time spent traveling to these locations, and reasons for traveling to specific locations over others. The data was collected by GfK Custom Research who administered this nationally representative public opinion survey to adults who were 18 years or older. All participants provided written consent.

Survey data collected through KnowledgePanel® perfectly matches the most current benchmarks of the Current Population Survey along several important demographic dimensions to be representative of the larger U.S. population, and post-stratification survey weights were included in the analysis to ensure U.S. population representation, thus providing a more generalizable results based upon demographic and socioeconomic variables. When potential respondents did not have adequate access to internet or a computer, GfK provided internet access and a laptop so that the individual could complete the survey. Detailed description of this survey administration and instrument items is available in previous research [15, 16]. The median survey completion time was 24 min and upon completion the respondents were entered into raffles for cash rewards and other prizes.

### Study instruments

The dependent variables of interest here examine different aspects of choice of where the individual purchases foods as well as the amount of time it takes for the respondent to travel to where they purchase foods. Each dependent variable measures a distinct component of access to fresh foods or factors that may complicate access to fresh foods. To illustrate, individuals who live in large cities and use public transportation may have difficulty transporting groceries home and instead choose to shop at gas stations, or other convenience stores. The difficulty accessing fresh fruits and vegetables may compromise their health over a number of years.

We examine 5 dependent variables that address food-related issues. Specifically, we ask respondents “When you buy food, how important is each of the following reasons for choosing where you purchase your food?” The survey items are: save money, drive less, better selection of food, and ability to buy organic foods. Each item is treated as its own dependent variable. The answers range from 1 to 5 where 1 = not important, 2 = somewhat important, 3 = important, 4 = very important, and 5 = extremely important. We also ask respondents “how long (in minutes) does it take you to travel to where you buy most of your food?” This is an open-ended question and we use the respondents written-in answer as another dependent variable.

The primary predictor variable of interest is racial minority status. This is a binary variable where white non-Hispanic = 0 and racial minority = 1. The binary variable is derived from the race categories noted in Table 1. In addition to minority status, we also control for several other socio-demographic characteristics that could influence food purchase choices. Household income is on an 11-point scale where 1 = less than $10,000 per year and 11 = greater than $125,000 per year. Education is on a 5-point scale where 1 = less than high school and 5 = professional degree. Conservative political ideology is a sliding scale that ranges from 1 to 7 where 1 = extremely liberal and 7 = extremely conservative. Age is the respondents reported age in years. Sex_Female is 1 = female and 0 = otherwise. Employment status is a binary variable where 1 = currently employed and 0 = otherwise. Finally, metropolitan statistical area (MSA) status captures whether the respondent live in a predominantly rural or metropolitan area, where 1 = metropolitan and 0 = otherwise.

**Table 1:** Descriptive Statistics: Predictor Variables

Characteristics	N (%)
**Sex**	
Male	798 (49.5)
Female	814 (50.5)
**Income**	
< $20,000	207 (12.85)
$20,000–$40,000	345 (21.41)
$40,000-75,000	406 (25.19)
> $75,000	654 (40.57)
**Race**	
Non-Hispanic White	1182 (73.33)
LatinX	164 (10.17)
African American	153 (9.49)
Other	52 (3.23)
**Age in Years**	
Mean (SD)	49.86 (17.30)
**Age in Groups**	
< 29	272 (17)
30–44	364 (23)
45–59	452 (28)
60+	524 (32)

Bivariate regression as well as multivariate were calculated and reported. We estimate an ordered logistic regression and an ordinary least squares regression, both with robust standard errors, to determine the relationship between sociodemographic characteristics and food choice characteristics. We use an ordered logistic regression because four of the dependent variables are bound and ranked from low to high. For the Minutes to Store variable we estimate and ordinary least square regression. Because of the skewed distribution of the Minutes to Store variable, we use a natural log transformation in the statistical regression.

## Results

A nationally representative sample was attempted as can be seen in Table 1. The response rate of 61.0% yielded a sample size of 1612 or 1612 completed surveys. There was rough parity between men and women and broad representation of differing incomes. While there was slight over representation of non-Hispanic white individuals (73%), minority status participants had sizable representation (26%), and a wide range of ages were present with a mean age of 49.86. Of the respondent’s 18.67% resided in a rural location (*n* = 301) and 81.33% (*n* = 1311) resided in a metropolitan region. This is aligned with national data collected by the US Census that also reveals 80% in urban and 20% in rural locations across the country [17].

Descriptive statistics for responses to the main reasons to travel further to find and acquire food is presented in Table 2. These 5 dependent variables represent the main drivers of food choice as revealed through this survey. Overall individuals’ priority in choosing the location to shop was to save money, followed by better selection of food, proximity to store, travel time, and finally ability to buy organic foods. This accounts for all individuals regardless of race, income, or age.

**Table 2:** Descriptive Statistics: Dependent Variables

	Mean	Std. Dev.	Min	Max
Save Money	3.80	1.06	1	5
Drive Less	3.02	1.17	1	5
Better Selection of Food	3.64	1.02	1	5
Ability to buy organic foods	2.18	1.27	1	5
Travel time in minutes (NL)	2.22	0.71	0	4.79

Results from the statistical analyses are presented in Table 3. As stated above, we are primarily interested in the relationships between the food security characteristics and minority status. As such we present results from a bivariate regression as well as multivariate, which includes the variables listed previously. We also interpret significant coefficients as odds ratios with robust standard errors, where appropriate.

**Table 3:** Bivariate and Multivariate Results Predicting Access to Foods

	Save Money		Drive Less		Better Selection		Buy Organic Foods		Minutes to Store	
Minority Status	1.306**	1.209	1.252*	1.132	1.343**	1.353**	1.918***	1.954***	0.139***	0.183***
	(0.144)	(0.144)	(0.132)	(0.130)	(0.149)	(0.161)	(0.201)	(0.227)	(0.040)	(0.042)
Household Income		0.925***		0.951**		1.022		1.006		0.001
		(0.016)		(0.016)		(0.018)		(0.017)		(0.006)
Education		0.925		0.961		0.986		1.166***		−0.039**
		(0.043)		(0.041)		(0.043)		(0.050)		(0.016)
Age		0.992**		1.001		1.004		0.988***		0.001
		(0.003)		(0.003)		(0.003)		(0.003)		(0.001)
Female		1.131		1.403***		1.242*		1.233*		0.061
		(0.108)		(0.134)		(0.117)		(0.120)		(0.035)
Employment Status		1.010		1.043		0.923		0.794*		−0.137***
		(0.109)		(0.111)		(0.102)		(0.086)		(0.039)
MSA Status		1.582***		1.446***		1.245		0.926		−0.298***
		(0.200)		(0.162)		(0.151)		(0.109)		(0.046)
Constant cut1	0.022*	0.015*	0.131*	0.092*	0.036*	0.044*	0.860*	0.463*		
	(0.004)	(0.005)	(0.011)	(0.026)	(0.005)	(0.014)	(0.049)	(0.123)		
Constant cut2	0.134*	0.100*	0.507*	0.365*	0.139*	0.181*	2.086*	1.146		
	(0.011)	(0.030)	(0.030)	(0.100)	(0.011)	(0.055)	(0.126)	(0.303)		
Constant cut3	0.698*	0.543*	2.238*	1.668	0.831*	1.098	6.187*	3.432*		
	(0.040)	(0.162)	(0.136)	(0.457)	(0.047)	(0.327)	(0.470)	(0.911)		
Constant cut4	2.180*	1.740	7.094*	5.301*	3.847*	5.118*	15.245*	8.421*		
	(0.129)	(0.518)	(0.556)	(1.503)	(0.252)	(1.541)	(1.545)	(2.353)		
Constant									2.185*	2.590*
									(0.020)	(0.102)
Wald Chi^2^	5.92***	61.49**	4.54***	52.34***	7.07***	27.62***	28.63***	86.87***		
R-squared									0.008	0.061
Pseudo R^2^	0.001	0.018	0.001	0.011	0.002	0.007	0.009			
F									12.05***	12.28***

*p < 0.05, ** *p* < 0.01, *** *p* < 0.001. (“Minutes to Store” has a coefficient presented, all others are presented as odds ratios. Robust standard errors in parentheses)

Across all bivariate models, where minority status is treated as the only covariate, we find that there is a positive and significant relationship. This suggests that minorities consider saving money, driving less, having a better selection of foods, and have the ability to buy organic foods as an important factor when choosing where to buy foods. Specifically, we find that minorities are approximately 30% more likely to think that saving money is important, 25.2% more likely to consider driving less as important, about 34% more likely to think selection of food is important, and nearly twice as likely (1.918) when considering the ability to buy organic food as important when compared to non-minorities in deciding where to purchase foods. Similarly, we find that minorities travel farther (in minutes) than non-minorities to the specific location where they buy their foods. This relationship is also statistically significant.

There was also a divide between urban and rural populations. While African American populations travel the furthers distance to acquire food, Hispanic groups travel the least distance.

## Discussion

This research suggests a desire within minority communities to have greater access to healthy food options in closer proximity to their homes. Further, minority populations are significantly (*p*-value < 0.001) more likely to have to drive further to arrive at these desired grocery stores. However, Hispanic groups did not see these same results. These findings provide insight on the importance of certain characteristics that remain prevalent when deciding where people in minority groups purchase food. Other research has found a similar result in rural areas in Texas in which Hispanic groups had better access to food sources than other minority populations [18] contrary to urban regions. One possible explanation for this is the rise of ethnic food marts and farmers markets catering to a rising population of Hispanic populations in the United States and points to a possible road map to improve food access to other groups and within urban areas.

This study has several important limitations. This utilized a cross-sectional study design in which characteristics of the predictor and dependent variables were collected simultaneously and does not allow for analysis over periods of time. As a result, we cannot, and do not imply any causal relationships here. Additional analyses that focus on decision-making processes regarding food purchase patterns and changes in one’s economic environment would illustrate important nuances to this argument. Further, data was self-reported and subjected to recall bias, previous research on dietary intake [19, 20], as well as estimates on time travelled [21] has found that both underestimate the time and amount consumed, this suggests that our results may be pulled toward the null hypothesis. As is typical with survey data, there is a possibility that qualitative difference in individuals’ lives may influence decisions. Additionally, some cultural differences are omitted in survey analysis. Finally, while many participants provided data, the response rate was low (61%). The results did benefit from a nationally representative sample and many important potentially confounding variables were collected. Furthermore, the results are consistent across multiple models and provide further evidence that food availability is unequal across racial category.

Diet and nutrition play a critical role in living a healthy life and are drivers of chronic health conditions and obesity levels [22]. It is becoming a public health crisis that is impacting the most vulnerable communities among us. While these results point towards an unequal access to healthy foods, implementation of food desert interventions need to operate with care. Research Sullivan (2014) found that when culturally appropriate grocery stores are not implemented in gentrified communities, minority populations fail to shop within them [23]. The findings from this research build upon previous work ad indicate at least one facet of this problem and may provide opportunities for future interventions and target polices.

## Conclusion

In this nationally representative sample, we found that minority populations travel further to reach grocery stores that served their needs and were more likely to consider organic food selection and price to be important factor when selecting a grocery store when compared to non-Hispanic white populations. This research furthers previous studies that have confirmed both an unequal access to affordable, nutritious food options [24], but also highlights individuals desire for better selections for them and their families within minority groups. The importance in creating programs and policies that address food insecurity is underscored by the reality that minority populations shoulder the largest burden of negative health conditions associated with a poor diet, linking their neighborhood characteristics to health implications.

## Supplementary Information

**Additional file 1.**

## Data Availability

Upon reasonable request data may be made available, please contact sansom@tamhsc.edu

## Abbreviations

GIS: Geographic Information Systems
WHO: World Health Organization
WEF: Water, Energy, Food
MSA: Metropolitan Statistical Area
