# Supplementary material for: Disparate access to nutritional food; place, race and equity in the United States
Source: BMC Nutr. 2021 Jun 29;7:29. doi: 10.1186/s40795-021-00434-2 (PMC8240216; doi:10.1186/s40795-021-00434-2)
Supplement: Supplementary file 1 — Additional file 1. [file 40795_2021_434_MOESM1_ESM.pdf]

Energy-Food-Water Nexus Public Survey - MAIN
August 5, 2015
- Questionnaire -

[DISPLAY]

Thank you for continuing to be part of the KnowledgePanel®. This research survey asks about your understanding of issues related to energy, food, and water resources and how they relate to each other, their risks, and your policy preferences. This survey is being conducted on behalf of the Institute for Science, Technology and Public Policy at Texas A&M University. The study will help researchers better understand how the U.S. public views these issues and their policy choices.

As with all KnowledgePanel® surveys, your response to this survey, or to any individual question on this survey, is completely voluntary. You will not be individually identified and your responses will be used only for analytical purposes to support scholarly research. The study involves one survey that should take between 22–25 minutes to complete.

If you have questions about your rights as a participant in this survey, or are dissatisfied at any time with any aspect of the survey, you may call the GfK Panel Member Support Center at 800-782-6899 or call the Texas A&M University Human Subjects Protection Program office at 855-795-8636 or email them at irb@tamu.edu.

By completing the survey, you are giving permission for the investigator to use your responses for research purposes.

IRB NUMBER: IRB2015-0324M
IRB APPROVAL DATE: 07/29/2015
IRB EXPIRATION DATE: 05/15/2020

[GLOBAL FORMATTING PROGRAMMING NOTE: PLEASE INCLUDE THE BELOW IMAGE ON EVERY SCREEN OF THE SURVEY FROM Q1-QF1 IN THE LOWER LEFT CORNER]

IRB NUMBER: IRB2015-0324M
IRB APPROVAL DATE: 08/10/2015
IRB EXPIRATION DATE: 05/15/2020

Proprietary

[General Concern, Governance & Knowledge Battery]

[GRID, SP ACROSS]
[RANDOMIZE Q1_A-Q1_K]

Q1. How concerned are you about each of the following issues?

Not at all Concerned										Extremely Concerned
0	1	2	3	4	5	6	7	8	9	10

a) Jobs and economic growth
b) Immigration
c) Pollution
d) Government spending/national debt
e) Global warming and climate change
f) Energy supply
g) Health care
h) Terrorism and national security
i) The environment
j) Water quality
k) Food availability

[GRID, SP ACROSS]
[RANDOMIZE Q2_A-Q2J]

Q2. How trustworthy are the following types of organizations?

Not at all Trustworthy									Extremely Trustworthy
0	1	2	3	4	5	6	7	8	9	10

a) Federal government
b) State government
c) Local government
d) Business corporations
e) Environmental groups
f) US Department of Agriculture
g) US Environmental Protection Agency
h) US Army Corps of Engineers
i) US Department of Energy
j) University scientists

[CREATE DOV_Q3A AND RANDOMLY ASSIGN R'S A VALUE OF 1 OR 2]
[GRID, SP ACROSS]

Q3a. Indicate what you think the appropriate role, or level of involvement, should be for the following types of organizations in **managing agricultural resources**.

No Role	Minor Role	Major Role	Lead Role

a) **[IF DOV_Q3A=1 INSERT:** Federal government**; IF DOV_Q3A=2 INSERT:** US Department of Agriculture**]**
b) State government
c) Local government
d) Private industry
e) Farmers
f) Nonprofit organizations

[GRID, SP ACROSS]

Q3b. Indicate what you think the appropriate role, or level of involvement, should be for the following types of organizations in **funding agricultural programs.**

Proprietary

No Role	Minor Role	Major Role	Lead Role

a) **[IF DOV_Q3A=1 INSERT:** Federal government**; IF DOV_Q3A=2 INSERT:** US Department of Agriculture**]**
b) State government
c) Local government
d) Private industry
e) Farmers
f) Nonprofit organizations

[CREATE DOV_Q4A AND RANDOMLY ASSIGN R'S A VALUE OF 1-3]
[GRID, SP ACROSS]

Q4a. Indicate what you think the appropriate role, or level of involvement, should be for the following types of organizations in **managing water resources**.

No Role	Minor Role	Major Role	Lead Role

a) **[IF DOV_Q4A=1 INSERT:** Federal government**; IF DOV_Q4A=2 INSERT:** US Army Corps of Engineers**; IF DOV_Q4A=3 INSERT:** US Environmental Protection Agency**]**
b) State government
c) Local government
d) Local or regional water authority
e) Private industry
f) Farmers
g) Nonprofit organizations

[GRID, SP ACROSS]

Q4b. Indicate what you think the appropriate role, or level of involvement, should be for the following types of organizations in **funding water programs**.

No Role	Minor Role	Major Role	Lead Role

a) **[IF DOV_Q4A=1 INSERT:** Federal government**; IF DOV_Q4A=2 INSERT:** US Army Corps of Engineers**; IF DOV_Q4A=3 INSERT:** US Environmental Protection Agency**]**
b) State government
c) Local government
d) Local or regional water authority
e) Private industry
f) Farmers
g) Nonprofit organizations

[CREATE DOV_Q5A AND RANDOMLY ASSIGN R'S A VALUE OF 1 OR 2]
[GRID, SP ACROSS]

Proprietary

Q5a. Indicate what you think the appropriate role, or level of involvement, should be for the following types of organizations in **managing energy resources**.

No Role	Minor Role	Major Role	Lead Role

a) **[IF DOV_Q5A=1 INSERT:** Federal government; **IF DOV_Q5A=2 INSERT:** US Department of Energy**]**
b) State government
c) Local government
d) Local or regional energy authority
e) Private industry
f) Energy producers
g) Nonprofit organizations

[GRID, SP ACROSS]

Q5b. Indicate what you think the appropriate role, or level of involvement, should be for the following types of organizations in **funding energy programs**.

No Role	Minor Role	Major Role	Lead Role

a) **[IF DOV_Q5A=1 INSERT:** Federal government; **IF DOV_Q5A=2 INSERT:** US Department of Energy**]**
b) State government
c) Local government
d) Local or regional energy authority
e) Private industry
f) Energy producers
g) Nonprofit organizations

[GRID, SP ACROSS]
[RANDOMIZE Q6_A-Q6_F]

Q6. Decide if each of these statements is true or false.

True	False

a) Using hydraulic fracturing to remove natural gas from the ground uses significant amounts of water.
b) Periods of drought can mean that an individual power plant cannot make as much electricity.
c) Corn used as ethanol fuel is the source of 10-15% of the energy consumed in the U.S.
d) Recycled water cannot be safely used to grow food.
e) Corn used as ethanol fuel gives cars better gas mileage than gasoline.
f) Crop irrigation in the U.S. uses more groundwater than all other uses combined.

[WATER BATTERY]

[SP]

Q7. What is your main source of household water?

Proprietary

1) Private well
2) Municipal water supply
3) Special district
4) Private company water supply

[IF Q7=2-4 OR REFUSED]
[SP]

Q8. Where does your water supplier get most of the water distributed to households?

1) From within the boundaries of the city or town itself
2) From outside the boundaries of the city or town itself
3) Not sure

[SP]

Q9. Have you personally been affected by drought in your area in the last three years?

1) Yes
2) No

[SP]

Q10. Have you personally been affected by flooding in your area in the last three years?

1) Yes
2) No

[IF Q7=2-4 OR REFUSED]
[SP]

Q11. Do you receive a water bill where you live?

1) Yes
2) No

[IF Q11=1 OR REFUSED]
[SP]

Q12. How often do you receive a water bill where you live?

1) Once every month
2) Once every three months (quarterly)
3) Once every year

[IF Q11=1 OR REFUSED]
[NUMBERBOX, RANGE 0-10000]

Q13. About how much is the average water bill you receive? $________

[IF Q7=2-4 OR REFUSED]
[GRID, SP ACROSS]

Q14. In the time that you have lived in your current residence, have you had any of the following problems with your water service?

Proprietary

Yes	No

a) The water does not taste good
b) The water is cloudy or dirty
c) Water pressure is low
d) The water causes sickness
e) Water billing or payment problems

[IF ANY IN THE Q14_A-Q14_E SERIES=1 "YES"]
[SP]

Q15. Did you contact your water utility to address any of the problems you experienced?

1) Yes
2) No

[IF Q15=1]
[SP]

Q15a. How would you describe your water utility's response to your concerns?

The utility:

1) Solved the problem completely and quickly
2) Solved the problem completely, but not quickly
3) Expressed interest in the problem but did not solve it
4) Ignored or dismissed the problem and did not solve it
5) Other

[IF Q15=2]
[SP]

Q15b. Why didn't you contact your water utility to address the problem or problems?

1) The problem wasn't severe enough.
2) I thought that the utility would solve the problem on its own.
3) Contacting the utility takes too much time or effort.
4) The utility doesn't care about people like me.
5) Other

[CREATE DOV_Q16 AND RANDOMLY ASSIGN R'S A VALUE OF 1-6. USE THE CHART BELOW FOR INSERTS]

DOV_Q16	Q16_INSERT_1	
1	$5 per month	
2	$10 per month	
3	$20 per month	
4	$60 per year	
5	$120 per year	
6	$240 per year	

[SP]

Proprietary

Q16. Would you be willing to pay more on your water bills to guarantee that there will be little to no flooding in your area? Would you be willing to pay **[Q16_INSERT_1]** on your water bill to guarantee that there will be little to no flooding in your area?

1) Yes
2) No

[GRID, SP ACROSS]
[RANDOMIZE Q17_A-Q17_N]
[INSERT BREAK IN GRID WITH SCALE DISPLAYED AGAIN AFTER 6TH QUESTION IN Q17_A-Q17_N SERIES]
[PUT A SPACE BETWEEN "CRITICALLY IMPORTANT COLUMN AND "DON'T KNOW" COLUMN]

Q17. Please rate the importance of the following water-related challenges on a scale of unimportant to critically important.

Unimportant	Slightly Important	Important	Very Important	Critically Important	Don't know

a) Watershed or source water protection
b) Water conservation / efficiency
c) Affordability of water service for low-income households
d) Groundwater management and overuse
e) Renewal and replacement of aging water and wastewater infrastructure
f) Long-term water supply availability
g) Storm water management and costs
h) Compliance with government regulation
i) Security of water utility infrastructure
j) Climate change risk
k) Loss of drinking water because of leakage from the system
l) The amount of electricity needed to pump water
m) Competition between farms and households for needed water
n) The amount of water needed to produce energy

[GRID, SP ACROSS]
[RANDOMIZE Q18_A-Q18_L]
[INSERT BREAK IN GRID WITH SCALE DISPLAYED AGAIN AFTER 6TH QUESTION IN Q18_A-Q18_LSERIES]

Q18. A number of policy options have been proposed to manage water resources. Please indicate your level of opposition or support for each of the following options.

Strongly Oppose	Oppose	Neutral	Support	Strongly Support

a) Build dams and reservoirs
b) Build pipelines to bring water from other regions
c) Conduct campaigns for voluntary water conservation
d) Give tax incentives for the installation of water-saving equipment
e) Develop a comprehensive national plan for allocating water across state borders
f) Require low water use landscaping
g) Require that lawn watering use reclaimed/reused water instead of drinking water

Proprietary

h) Give tax incentives for implementing efficient irrigation systems for agriculture
i) Build desalination plants to make sea water drinkable
j) Buy water from farmers to use in cities
k) Charge higher water rates during the hottest part of the summer
l) Charge higher water rates for high volume users

[ENERGY BATTERY]

[NUMBERBOX RANGE 0-10000]

Q19. About how much is your average monthly energy bill (for electric, for gas, or for the total of both electric and gas if you pay for both)?

$ [ ]

[SPACE]

1) I do not receive a monthly energy bill. **[SP]**

[GRID, SP ACROSS]

Q20. Some ways of generating electricity may be harmful to water supplies because they use significant amounts of water or create water pollution. How harmful to water supplies do you think each of these electrical power sources is?

Not Harmful	Somewhat Harmful	Harmful	Very Harmful	Extremely Harmful

a) Coal
b) Nuclear
c) Natural Gas
d) Hydroelectric
e) Solar
f) Wind
g) Waste to Energy
h) Biofuel, such as ethanol

[GRID, SP ACROSS]

Q21. Do you favor an increase or decrease in the use of the following energy sources over the next 25 years?

Increase	Stay the Same	Decrease

a) Coal
b) Nuclear
c) Natural Gas
d) Hydroelectric
e) Solar
f) Wind
g) Waste to Energy
h) Biofuel, such as ethanol

[CREATE DOV_Q22 AND RANDOMLY ASSIGN R'S A VALUE OF 1-3]

[GRID, SP ACROSS]

Proprietary

Q22. Rate how important each of the following criteria should be when building a new **[IF DOV_Q22=1 INSERT:** coal power plant**; IF DOV_Q22=2 INSERT:** nuclear power plant**; IF DOV_Q22=3 INSERT:** natural gas power plant**]** in your community.

0	1	2	3	4	5	6	7	8	9	10
Not at all Important										Extremely Important

a) Safety
b) Construction cost
c) Effect on the environment
d) Reduction of your energy bill
e) Creation of jobs
f) Proximity to your residence

[SP]

Q23. How much, if anything, have you heard about hydraulic fracturing, also known as fracking, a process that is used to extract oil and natural gas from underground rock formations?

1) A Lot
2) A Little
3) Nothing at all

[IF Q23=1 OR 2 OR REFUSED]
[GRID, SP ACROSS]

Q24. Do you agree or disagree with the following statements regarding water use for hydraulic fracturing, or fracking?

Strongly Disagree	Disagree	Neither Disagree Nor Agree	Agree	Strongly Agree

a) We should have policies requiring full disclosure of **chemicals used in fluids** for hydraulic fracturing.
b) We should have policies requiring full disclosure of **source water** used for hydraulic fracturing.
c) We should have more stringent regulations for the **disposal of fluids** from hydraulic fracturing.
d) We should have more monitoring of **groundwater** before and after hydraulic fracturing.

[IF Q23=1 OR 2 OR REFUSED]
[GRID, SP ACROSS]

Q25. Some people believe the following items are problems caused by hydraulic fracturing, or fracking. Please indicate your assessment of the seriousness of each item using the scale below.

Not a Problem at All									Extremely Serious Problem
0	1	2	3	4	5	6	7	8	9	10

a) Competition for water supplies for hydraulic fracturing.
b) Depletion of water sources (streams, groundwater, etc.) used in hydraulic fracturing.
c) Contamination of water sources from oil and gas drilling using hydraulic fracturing.

Proprietary

d) Contamination of water sources from the disposal of fluids used in hydraulic fracturing.

[GRID, SP ACROSS]
[RANDOMIZE Q26_A-Q26_L]
[INSERT BREAK IN GRID WITH SCALE DISPLAYED AGAIN AFTER 6TH QUESTION IN Q26_A-Q26_L SERIES]

Q26. A number of policy options have been proposed to manage energy resources. Please indicate your level of opposition or support for each of the following options.

Strongly Oppose	Oppose	Neutral	Support	Strongly Support

a) Build additional power plants
b) Build pipelines to bring oil from other regions
c) Conduct campaigns for voluntary energy conservation
d) Give tax incentives to energy companies to develop more solar energy technologies
e) Develop a comprehensive national plan for allocating energy across state borders
f) Relax environmental standards for energy industries
g) Require that new construction meets high energy efficiency standards
h) Give tax cuts to energy companies to increase oil and gas exploration in the US
i) Build charging stations for electric vehicles at government owned or funded facilities
j) Increase federal funding for research on renewable energy technologies
k) Charge higher energy rates during high demand times of day
l) Charge higher energy rates for high volume users

[FOOD BATTERY]

[NUMBERBOX]

Q27. How long (in minutes) does it usually take you to travel to where you buy most of your food?

[ ] minutes.

[SP]

Q28. When you go to a grocery store, how do you usually get there?

1. Drive myself
2. A friend drives me
3. Walk
4. Taxi
5. Bus, subway, or train
6. Other (please specify) **[TEXTBOX]**

[GRID, SP ACROSS]

Q29. When you buy food, how important is each of the following reasons for choosing where you purchase your food?

Not Important	Somewhat Important	Important	Very Important	Extremely Important

Proprietary

a) Save money
b) Drive less
c) Better selection of food
d) Able to buy organic food

[GRID, SP ACROSS]
[RANDOMIZE Q30_A-Q30_K]
[INSERT BREAK IN GRID WITH SCALE DISPLAYED AGAIN AFTER 6TH QUESTION IN Q30_A-Q30_K SERIES]

Q30. How concerned are you about each of the following issues related to agricultural production?

Not Concerned	Somewhat Concerned	Concerned	Very Concerned	Extremely Concerned

a) The ability of food crops to tolerate drought.
b) The ability of food crops to tolerate pests and disease.
c) The loss of productive crop lands due to the growth of urban areas.
d) The availability of fertile top soil.
e) The diversity of plant seed varieties to preserve genetic material.
f) The amount of herbicides, pesticides, and fertilizers used in food production.
g) The amount of food wasted by grocery stores, restaurants, and cafeterias.
h) The nutritional quality of the food produced.
i) The amount of energy used to produce the food.
j) The amount of water used to produce the food.
k) Water pollution caused by farms.

[GRID, SP ACROSS]
[RANDOMIZE Q31_A-Q31_L]
[INSERT BREAK IN GRID WITH SCALE DISPLAYED AGAIN AFTER 6TH QUESTION IN Q31_A-Q31_L SERIES]

Q31. A number of policy options have been proposed to manage agricultural resources. Please indicate your level of opposition or support for each of the following options.

Strongly Oppose	Oppose	Neutral	Support	Strongly Support

a) Provide space free of charge for community gardens
b) Build composting facilities for food waste
c) Conduct campaigns to encourage buying locally grown foods
d) Give tax incentives for farmers to reduce the use of fertilizers and pesticides
e) Develop a comprehensive national plan for preserving our agricultural lands
f) Require that farmers use soil conservation measures
g) Limit the amount of land that can be used to grow crops for biofuels rather than food
h) Give tax incentives for farmers to use more energy efficient methods of growing and transporting food
i) Provide space free of charge for local farmers' markets
j) Establish seed banks to maintain biodiversity of critical food crops
k) Charge significant impact fees to housing developers to help prevent the loss of agricultural land
l) Charge higher licensing fees to restaurants that do not follow an approved plan to reduce food waste

[CREATE DOV_Q32 AND RANDOMLY ASSIGN R'S A VALUE OF 1 OR 2]

Proprietary

[MP]
[RANDOMIZE Q32_A-Q32_I]

Q32. Have you or has someone in your household done any of the following to conserve **[IF DOV_Q32=1 INSERT:** water**; IF DOV_Q32=2 INSERT:** energy**]**?

a) Installed low-flow fixtures.
b) Routinely take shorter showers.
c) Routinely use the dish washer less often and/or with fuller loads.
d) Routinely use the washing machine less often and/or with fuller loads.
e) Installed a more efficient hot water heater.
f) Installed more efficient appliances (washing machine, dishwasher).
g) Routinely compost household food waste.
h) Routinely drink beverages from a reusable container.
i) Installed a solar power system for some household applications.
j) None of the above **[ANCHOR LAST; EXCLUSIVE CHOICE]**

[SP]
Q33. Are you a member of a group that promotes renewable energy?

1) Yes
2) No

[SP]
Q34. Do you participate in a food co-op or community garden group?

1) Yes
2) No

[SP]
Q35. Are you a member of a group that promotes water conservation?

1) Yes
2) No

[INSERT STANDARD CLOSE, NOTED BELOW FOR REFERENCE]

[TEXTBOX]
QF1. Thinking about this topic, do you have any comments you would like to share?

[DISPLAY]
Thank you for completing this survey. We have successfully received your responses.

Proprietary
